# Relationship between raltegravir trough plasma concentration and virologic response and the impact of therapeutic drug monitoring during pregnancy

**DOI:** 10.1177/09564624221144489

**Published:** 2022-12-18

**Authors:** Sabrina Carvalho, Nancy L. Sheehan, Silvie Valois, Fatima Kakkar, Marc Boucher, Ema Ferreira, Isabelle Boucoiran

**Affiliations:** 1 Faculty of Pharmacy, Ringgold: 63677 Université de Montréal, Montréal, QC, Canada; 2 Mother and Children’s Infectious Diseases Center, Ringgold: 70443 Centre Hospitalier Universitaire Sainte-Justine, Montréal, QC, Canada; 3 Pharmacy Department and Chronic Viral Illness Service, Ringgold: 54473 McGill University Health Centre, Montréal, QC, Canada; 4 Department of Pediatrics, Ringgold: 5622 Université de Montréal, Montréal, QC, Canada; 5 Department of Obstetrics and Gynecology, Ringgold: 5622 Université de Montréal, Montréal, QC, Canada; 6 Department of Pharmacy, Ringgold: 70443 Centre Hospitalier Universitaire Sainte-Justine, Montréal, Québec, Canada; 7 School of Public Health, Ringgold: 5622 Université de Montréal, Montréal, QC, Canada

**Keywords:** Raltegravir, therapeutic drug monitoring, pregnancy, HIV

## Abstract

**Background:**

Limited data is available on raltegravir (RAL) pharmacokinetics during pregnancy and the value of therapeutic drug monitoring (TDM) in pregnancy is unknown. This study aims to describe RAL trough plasma concentrations (C_trough_) during pregnancy and review the impact of RAL TDM on outcomes.

**Methods:**

Women from the prospective mother-infant HIV cohort of Mother and Children’s Infectious Diseases Center who received RAL during their pregnancy between 2011-2020 were included. TDM reports were reviewed and C_trough_ values estimated when possible, using historical RAL half-lives.

**Results:**

We included 76 pregnant women of which 47 underwent TDM. We observed a significant association between virological response and C_trough_ (*p*-value .034) with an increase of 0.1 mg/L corresponding to a 2.96 reduction in the risk of having a detectable viral load. The results indicated that in pregnant women a RAL C_trough_ threshold of 0.04 mg/L has a higher specificity (75%) as compared to our current C_trough_ target value of 0.02 mg/L (25%) and an acceptable sensitivity (77%). No significant differences were observed between C_trough_ at each trimester. When comparing pregnancies with and without TDM, no statistically significant differences were observed in the virologic response during pregnancy and at delivery, or with the need for triple antiretroviral prophylaxis in newborns.

**Conclusions:**

An association between RAL C_trough_ and viral load was observed and achieving a RAL C_trough_ of 0.04 mg/L or greater is a predictor of virologic response in pregnant women. The impact of TDM in pregnancy, however, could not be demonstrated.

## Introduction

Antiretroviral therapy (ART) during pregnancy in women living with HIV is important for the health of both mother and newborn. ART can reduce maternal viral loads to below the limit of detection, thereby decreasing the risk of mother-to-child transmission from 15 to 45% to less than 1% in high income countries.^1^ Several guidelines recommend integrase inhibitors, including raltegravir (RAL), as first-line treatment during pregnancy and for women of childbearing age.^2,3^ A population pharmacokinetic model to explore RAL pharmacokinetic changes during pregnancy has been described,^4^ and three clinical studies have investigated the pharmacokinetics of RAL in pregnant women living with HIV.^5–7^ These studies reported a mean decrease of 28 % or greater in RAL plasma concentrations (C_p_) during the third trimester relative to postpartum. These studies were, however, limited by small sample sizes. Additionally, RAL high dose (HD) 1200 mg once daily dosing is not recommended during pregnancy by international guidelines due to limited pharmacokinetic data.^2,8^

Subtherapeutic plasma ART levels can increase the risk of maternal viral resistance to treatment, and HIV transmission from mother to fetus,^9^ while overexposure can result in embryo-fetal and maternal toxicity.^10^ Some health centers have implemented therapeutic drug monitoring (TDM) protocols during pregnancy to ensure that C_p_ of ART are maintained in the therapeutic range, and results have been equivocal.^11–13^ The usefulness of TDM of RAL in pregnancy has not been studied.^11^

The primary objective of this study was to determine RAL trough plasma concentrations (C_trough_) in pregnancy. Secondary objectives were to investigate whether TDM had a positive impact on virologic response in pregnancy and on the need, or not, to use a triple combination antiretroviral prophylaxis for the newborn.

## Methods

### Study design and participants

This study uses data from the prospective mother-infant HIV cohort of the Women and Children’s Infectious Diseases Center (CIME) in Montreal, Canada.^14^ This cohort has been recruiting women in first trimester pregnancy living with HIV since 1987. The present study was approved by the CHU Sainte-Justine research ethics committee.

Adult women who received RAL at any time during pregnancy and whose delivery took place between 1 January 2011, and 1 August 2020, at CHU Ste-Justine were included in our analyses. Cases of first trimester spontaneous or voluntary abortion (< 14 weeks) were excluded. For women that were pregnant more than once during the study period, only the most recent pregnancy was considered.

### Therapeutic drug monitoring

TDM is recommended *as per* HIV clinical and antiretroviral TDM guidelines for the province of Quebec at each trimester during pregnancy.^15,16^ Blood samples are analyzed and interpreted by the Québec Antiretroviral Therapeutic Drug Monitoring Program of the McGill University Health Center (MUHC), Montréal, Canada^16^

Samples were collected in a non-gel heparinized tube and plasma was obtained by centrifugation at 3000g for 5 min and transferred to polypropylene cryotubes which were stored at −20°C until analyses. RAL C_p_ was determined by liquid chromatography tandem mass spectrometry (LC/MS/MS). This method was validated by two external quality control programs (KKGT, the Netherlands; Asqualab, France). The limit of detection of this assay was 0.01 mg/L with an intra-assay and inter-assay coefficient of variability of less than 6.2% and 8%, respectively. When RAL C_p_ were below the limit of detection, a C_trough_ of 0.005 mg/L was assigned.

For the secondary objective, women were divided into two groups: a TDM group, including women with at least one interpretable RAL TDM result during the pregnancy, and the “No TDM” group which included women without interpretable TDM or who had no TDM done during their pregnancy. A TDM was designated as interpretable based on the concentration at the end of the dosing interval (C_trough_) or, if the C_trough_ was not available, on pharmacist expertise.

### Data collection

Patient demographics, medical histories, laboratory and clinical data, and mother’s and infant’s ART histories were extracted from the CIME cohort database. Virologic response was defined as a viral load of < 50 HIV RNA copies/mL.

Hepatotoxicity was determined from serum alanine aminotransferase (ALT) levels. The last recorded ALT level prior to TDM was categorized according to the U.S. Department of Health and Human Services Adverse Event Severity Classification Table.^17^

TDM interpretation reports were reviewed and data on RAL C_p_, time post-dose of sample procurement, and pharmacist interpretations and recommendations were extracted. When blood samples were not taken exactly at the end of the dosing interval but at least 8 h post-dose for twice daily dosing and 12 h post-dose for once daily dosing, the C_trough_ were estimated using historical population RAL half-life values^6,18,19^ as presented in Supplementary Figure 1. The treshold used to determine therapeutic status was RAL C_trough_ ≥ 0.02 mg/L.^16,20^ If the sample was not taken at the end of the dosing interval or a C_trough_ extrapolation was not possible as the sample was taken too early in the dosing interval (i.e., < 8 h post-dose for twice daily dosing and < 12 h post-dose for twice daily dosing), the TDM pharmacist could determine if the result was subtherapeutic or therapeutic based on expert opinion and by comparing the result to the mean population curve.

Possible causes for subtherapeutic RAL C_trough_ obtained from patient medical charts were classified as: 1) suboptimal adherence to treatment, 2) drug interactions, and 3) unknown, and therapeutic decisions made by treating physicians were: 1) RAL dose adjustments, 2) encouraging adherence, 3) changes in ART 4) changing/adjusting concomitant medications, and 5) other.

### Statistical analysis

The relationship between RAL C_trough_ and virologic response throughout pregnancy was analyzed using a generalized estimation equation (GEE) to account for women who had multiple C_trough_ determinations. A receiver operating characteristic (ROC) curve analysis including 95% confidence intervals (95% CI) was performed to determine the RAL threshold C_trough_ value that best predicts a virologic response.^21^ Median RAL C_trough_ were compared between each trimester and to a historical median RAL C_trough_ in non-pregnant adult women living with HIV (median RAL C_trough_ = 0.093 mg/L).^22^ Mean C_trough_ was used for women with multiple assessments in the same trimester.

The value of RAL TDM in pregnancy was evaluated by comparing the ‘TDM’ and ‘No TDM’ groups association for 3 outcomes: 1) proceeding to triple combination antiretroviral prophylaxis in the newborn, 2) virologic response during the whole pregnancy, and 3) virologic suppression closest to delivery. The first outcome relates to the use since 2011 of a combination of three antiretrovirals (zidovudine, lamivudine and nevirapine or RAL) in newborns at high risk of infection (i.e., detectable maternal viral load immediately prior to or at the time of delivery, < 4 weeks of ART prior to delivery, suspicion of/known non-adherence in pregnancy, and persistently detectable viremia during pregnancy)^15^ at CHU Sainte-Justine. Associations were determined using the Fisher’s exact test or GEE. Characteristics between the two groups were compared using the Chi Square test of proportions or Mann-Whitney U Test as appropriate.

Statistical analyses were performed using SPSS statistical software, version 26 (IBM Corp, NY, USA). A *p*-value of < 0.05 was considered statistically significant.

## Results

### Cohort characteristics

Of the 746 women enrolled in the CIME cohort, 76 (10%) met the eligibility criteria for this study. The demographic and clinical characteristics of the study population are presented in Table 1. Half and 32% of the women were originally from Africa or the Caribbean, respectively. The mean age at childbirth was 33 years (± 5 years). There were 78 newborns (two twin pregnancies): 74 were confirmed HIV negative and 4 were of undetermined HIV status at last hospital follow-up.

**Table 1.** Demographic and clinical characteristics of women.^a^

	Total (*n* = 76)	TDM^b^ (*n* = 47)	No TDM^b^ (*n* = 29)	Missing	*p*-value^c^
**Age at delivery, years**	33 (5)	33 (5)	32 (6)	0	0.456
**Weight at delivery, kg**	85.8 (19)	84 (21)	85 (15)	1	0.730
**CD4+ lymphocyte count at delivery, cells/mm** ^**3**^	579 (299)	468 (322)	549 (253)	13	0.829
**CD8** ^**+**^ **lymphocyte count at delivery, cells/mm** ^**3**^	771 (390)	728 (305)	824 (531)	12	0.920
**Gestational age at delivery, weeks**	39 (1)	39 (2)	39 (1)	0	0.349
**Geographic origin**				0	
Africa	38 (50%)	23 (49%)	15 (52%)		1
Caribbean	24 (32%)	13 (28%)	11 (38%)		0.447
Other	14 (18%)	11 (23%)	3 (10%)		0.225
**ART naïve at conception**	36 (49%)	22 (47%)	14 (52%)	2	0.810
**Substance use during pregnancy**				0	
Recreational drugs (other than marijuana)	1 (1%)	1 (2%)	0		1
Cigarettes	9 (12%)	4 (9%)	5 (17%)		1
Marijuana	4 (5%)	3 (6%)	1 (3%)		0.633
Alcohol	7 (9%)	5 (11%)	2 (7%)		0.702
**Comorbidities**				0	
Diabetes	15 (20%)	7 (15%)	8 (28%)		0.556
Chronic hepatitis B	1 (1%)	1 (2%)	0		1
Hyperemesis gravidarum	1 (1%)	1 (2%)	0		1
**Concomitant antiretrovirals in third trimester**				0	
Two or more NRTIs only	62 (82%)	39 (83%)	23 (79%)		0.765
NRTIs plus PI	9 (12%)	5 (11%)	4 (14%)		0.725
PI	1 (1%)	1 (2%)	0		1
Other	5 (7%)	3 (6%)	2 (7%)		1
**Non-HIV medications during pregnancy**					
Proton pump inhibitor	6 (8%)	4 (9 %)	2 (7 %)	0	1
H_2_ receptor blockers	23 (30%)	12 (26 %)	11 (38 %)	0	0.308
Vitamins and minerals	62 (86%)	41 (87%)	21 (84%)	4	0.730

^a^Mean (SD) or number (%).

^b^the TDM group includes women with at least one interpretable RAL TDM result during pregnancy, while the No TDM group includes women without interpretable TDM or who had no TDM done during their pregnancy.

^c^Chi Square test of proportions or Mann-Whitney U Test.

Abbreviations: ART: Antiretroviral therapy; NRTI: nucleoside/nucleotide reverse transcriptase inhibitor; PI: protease inhibitor; TDM: Therapeutic drug monitoring.

There were 84 interpretable TDM results from 47 women. The other 29 women did not undergo TDM (21%) or TDM was uninterpretable (17%). All the TDM considered uninterpretable were taken too early or too late in the dosing interval to be able to extrapolate the RAL C_trough_. Of the 84 TDM results, 56 (67%) could be extrapolated to C_trough or_ C_trough_ was directly available. C_trough_ results grouped according to RAL dose are shown in Table 2. Overall, 15% of women had viral loads ≥ 50 copies/mL at the last assessment prior to TDM. No abnormal ALT levels were observed.

**Table 2.** Raltegravir concentrations and virologic efficacy in 47 women with therapeutic drug monitoring.

	Total no. TDM samples (*n* = 84)	RAL 400 mg twice daily TDM samples (*n* = 77)	RAL HD 1200 mg once a day TDM samples (*n* = 2)	RAL other dosage TDM samples^a^ (*n* = 5)	Missing
Median C_trough_ RAL concentration,^b^ mg/L(IQR)	0.07 (0.04–0.15)	0.08 (0.04–0.16)	0.01 (−)^c^	0.04 (0.04–0.04)^c^	28
Interpretation of RAL C_trough_, n (%)					0
Subtherapeutic	13 (15%)	11 (14%)	1 (50%)	1 (20%)	
Therapeutic	71 (85%)	65 (84%)	1 (50%)	4 (80%)	
HIV viral load before or at time of TDM (copies/mL), n (%)					0
<50	72 (86%)	66 (86%)	2 (100%)	4 (80%)	
50 - 200	7 (8%)	6 (8%)	0	1 (20%)	
>200	5 (6%)	5 (6%)	0	0	
Mean time between viral load measurement and TDM, days (SD)	4 (13)	3 (13)	0	8 (18)	

^a^RAL other dosages represent three RAL 800 mg twice daily TDM samples and two RAL 800 mg once daily TDM samples.

^b^C_trough_ were estimated or available for 56 TDM samples (see Supplementary Figure 1).

^c^The IQR is not possible in the RAL HD 1200 group since only one C_trough_ value was available. The four available Ctrough values for the RAL other dosage group were equal to 0.04 mg/L.

Abbreviations: Ctrough, concentration at the end of the dosing interval; HD, high dose formulation (600 mg tablet); RAL, raltegravir; TDM, therapeutic drug monitoring.

### RAL C_trough_ during pregnancy

**Figure 1.**
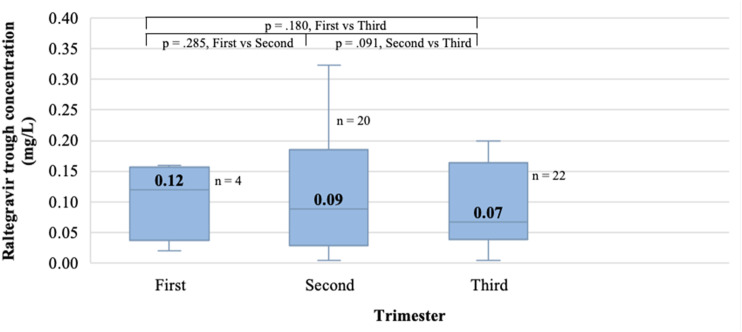
Evolution of the median raltegravir trough concentration according to the pregnancy trimester for raltegravir 400 mg twice daily. Legend: Mean C_trough_ was used for women with multiple assessments in the same trimester. This resulted in a total of 46 C_trough_ values. C_trough_ results for each trimester are presented with the median, the interquartile range, and the minimum and maximum values for each group. Outliers are not shown. *p*-values represent the comparison between trimesters in C_trough_ values and were determined by the related samples Wilcoxon signed rank test. Abbreviations: C_trough_, concentration at the end of the dosing interval; RAL, raltegravir.

Because most women received RAL 400 mg twice daily, this regimen was used to determine RAL C_trough_ throughout pregnancy. As shown in Figure 1, no statistically significant differences were observed in RAL C_trough_ from one trimester to another, and while the median C_trough_ (0.07 mg/L) during the third trimester was 24.7% lower than a historical cohort of non-pregnant women,^22^ this decrease was not statistically significant (*p*-value .067).

### Subtherapeutic C_trough_, possible causes and interventions

Subtherapeutic RAL TDM were observed in 13 (15%) of the 84 interpretable TDM results and 54% of these were attributed to non-adherence to ART. Encouraging adherence to treatment was the primary intervention (69%) reported by physicians (Table 3). Details on subtherapeutic TDM results and interventions done for each woman are available in Supplementary Table 1. The GEE demonstrated a significant association between virological response and C_trough_ (p -value .034) with an increase in C_trough_ of 0.1 mg/L corresponding to a 2.96 reduction in the risk of having a detectable viral load. The ROC curve demonstrated a good performance of the C_trough_ to predict virologic response (area under the curve: 0.814 [95% CI: 0.665–0.963]). In our cohort of pregnant women, a C_trough_ of less than 0.04 mg/L provided a higher specificity (sensitivity: 77% [95% CI 66.7–86.5] and specificity: 75% [95% CI 32.9–117.1]) than the current C_trough_ target of 0.02 mg/L (sensitivity: 92% [95% CI: 82.9–100.1] and specificity: 25% [95% CI: −16.8–66.8]), and an acceptable sensitivity.

**Table 3.** Suspected causes of subtherapeutic raltegravir therapeutic drug monitoring results and resulting therapeutic action.^a^

	Total no. subtherapeutic C_p_ (*n* = 13)	RAL 400 mg twice daily (*n* = 11)	RAL HD 1200 mg once a day (*n* = 1)	RAL 800 mg twice daily (*n* = 1)
**Suspected cause**				
Suboptimal adherence	7 (54%)	5 (45 %)	1 (100%)	1 (100%)
Drug interaction	1 (8%)	1 (9 %)	0	0
Unknown	5 (38%)	5 (45 %)	0	0
**Implemented intervention**				
Changes in ART	0	0	0	0
RAL dose adjustments	2 (15%)	2 (18%)	1 (100%)	0
Changing/adjusting interacting concomitant medication	1 (8%)	1 (9 %)	0	0
Encouraging adherence	9 (69%)	7 (64 %)	0	1 (100%)
Other	1 (8%)	1 (9 %)	0	0

^a^From *N* = 84 interpretable therapeutic drug monitoring (TDM) results.

Abbreviations: ART, antiretroviral therapy; Cp, plasma concentration; Ctrough, concentration at the end of the dosing interval; HD, high dose formulation (600 mg tablet); RAL, raltegravir; TDM: therapeutic drug monitoring.

### Association between therapeutic drug monitoring use and HIV-related outcomes

When evaluating the association between virologic response during pregnancy and TDM groups, no statistically significant associations were found for virologic response during the whole pregnancy (*p*-value .229) nor for virologic suppression closest to childbirth (*p*-value .549). In our cohort of neonates, 6 (21%) children from the ‘No TDM’ group received a triple antiretroviral prophylaxis regimen versus 9 (19 %) in the ‘TDM’ group. No statistically significant association was found for the use of this type of prophylaxis (*p*-value 1.0).

## Discussion

In our cohort of pregnant women, a RAL C_trough_ threshold of 0.04 mg/L obtained from ROC analysis had a higher specificity to predict virologic response (viral load < 50 copies/mL) than the currently used C_trough_ threshold of ≥ 0.02 mg/L. We favor a higher specificity to limit false positives, that is falsely identifying a person as at risk of virologic failure and unnecessarily increasing doses, costs, pill burden and potentially increasing adverse effects. The association between viral suppression and RAL C_trough_ was demonstrated in the QDMRK trial of a population of non-pregnant adults that received RAL 800 mg once daily and presented lower RAL C_p_.^23^ The C_trough_ threshold of 0.02 mg/L (45 nM) was then proposed from the ROC analyses in Rizk et al. where the C_trough_ threshold predictive performance (sensitivity 45% and specificity 75%) was lower than that found in our study (RAL C_trough_ 0.04 mg/L: sensitivity 77% and specificity 75%).^20^ Garrido et al. also studied the association between virologic failure and low RAL C_trough_ levels and reported a positive association between them but did not report a C_trough_ threshold value.^24^ This higher C_trough_ threshold found in our study could possibly be explained by changes in host immunity occurring during pregnancy and immune responses to infection that have been shown to depend on the trimester and the stage of infection.^25–27^ A compromised immune system at any time during pregnancy could possibly necessitate maintaining higher RAL C_trough_ for equivalent viral suppression. This hypothesis warrants further investigation.

More than half of the subtherapeutic TDM RAL C_trough_ were attributed to suboptimal adherence to medication. This demonstrates the challenges faced by people living with HIV and the need for continued support to facilitate access and promote adherence to ART. Some of the causes for the other subtherapeutic RAL C_trough_ concentrations remain unknown but could be due to physiologic or metabolic changes occurring in pregnancy, as evidenced by the 24.7% decrease in RAL C_trough_ in the third trimester compared to the C_trough_ of non-pregnant women, consistent with other reports.^4–7^ Regarding the RAL HD once daily dosing, we only had two TDM results, one of which was subtherapeutic. Even though the probable cause of this subtherapeutic result was poor adherence, physiological changes occurring during pregnancy could have contributed. Recently, a population PK study of RAL once-daily pooling 11 PK studies simulated a mean decrease in C_trough_ of 49% during pregnancy.^28^ These results further emphasize that this dosing regimen may not be appropriate for this patient population.

Though current Quebec provincial antiretroviral TDM guidelines moderately recommend RAL TDM in pregnancy, in our study only 77% of women had a RAL TDM and 62% of women had at least one interpretable RAL TDM.^15,16^ This highlights the difficulty of implementing TDM in real life even when it is easily accessible. The European AIDS Clinical Society (EACS), the British HIV Association (BHIVA) and the Department of Health and Human Services (DHHS) state to consider TDM of ART during pregnancy in specific clinically relevant situations.^2,3,29^ An update to the Québec TDM guidelines will include that RAL TDM is optional in pregnant women receiving the twice daily dose, but recommended in specific cases (e.g., detectable viral load, drug interactions, suspected malabsorption, etc.). If pregnant women receive the once-daily regimen despite it not being recommended, RAL TDM will remain strongly recommended given the limited data and the risk of subtherapeutic levels.

Hepatitis and hepatic failure are uncommon adverse reactions to RAL, and grade 2 or 3 serum ALT elevations have been reported.^30^ A case of high serum transaminase levels has been reported after adding RAL to an ART regimen in a pregnant woman.^31^ Although we did not observe elevations in serum ALT levels, liver function assessments in pregnant women receiving RAL remain warranted.

In addition to the small sample size in this study, other limitations should be considered. For instance, RAL C_trough_ values were obtained by extrapolation based on the RAL elimination half-life in 38% cases. To counterbalance this limitation, we carried out the extrapolation using half-lives reported in pregnancy. In addition, the limited number of subjects that received the once daily RAL regimen precluded analysis of the association between C_trough_ levels and virologic response in this group. As treating physicians may request TDM when poor patient adherence to medication or high risk of virologic failure due to resistance is suspected, a selection bias in the TDM versus the “No TDM” group is likely. We observed a low rate of virological failure in our cohort which did not allow us to carry out multivariate analysis of the association between C_trough_ levels and virologic response.

A RAL C_trough_ target of 0.04 mg/L was a predictor of virologic response for pregnant women in our trial. The target C_trough_ proposed in this study could improve the clinical relevance of RAL TDM remains to be validated in a larger study. In addition, further studies are needed to characterize the pharmacokinetics and safety of newer integrase inhibitors, such as cabotegravir and bictegravir, in pregnancy.

## Supplemental Material

Supplemental material - Relationship between raltegravir trough plasma concentration and virologic response and the impact of therapeutic drug monitoring during pregnancy Supplemental material for Relationship between raltegravir trough plasma concentration and virologic response and the impact of therapeutic drug monitoring during pregnancy by Sabrina Carvalho, Nancy L. Sheehan, Silvie Valois, Fatima Kakkar, Marc Boucher, Ema Ferreira and Isabelle Boucoiran in International Journal of STD & AIDS
