# Supplementary material for: Relationship between raltegravir trough plasma concentration and virologic response and the impact of therapeutic drug monitoring during pregnancy
Source: Int J STD AIDS. 2022 Dec 18;34(3):175–82. doi: 10.1177/09564624221144489 (PMC9925909; doi:10.1177/09564624221144489)
Supplement: Supplemental material - Relationship between raltegravir trough plasma concentration and virologic response and the impact of therapeutic drug monitoring during pregnancy [file sj-pdf-1-std-10.1177_09564624221144489.pdf]

Relationship between raltegravir trough plasma concentration and virologic response and the impact of therapeutic drug monitoring during pregnancy

Supplementary material

Supplementary Figure 1: Decision tree for extrapolation of raltegravir trough plasma concentrations from plasma concentrations

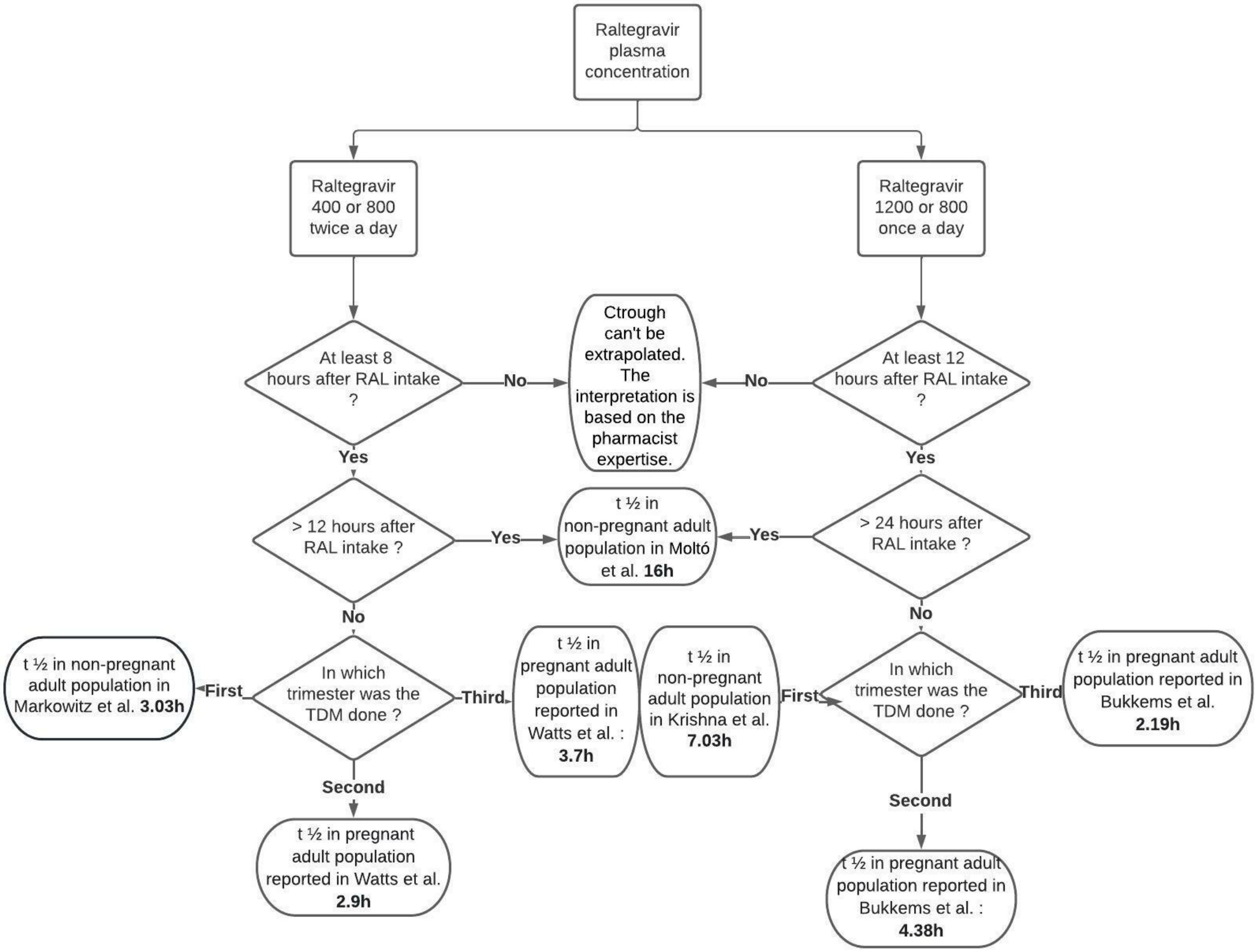

Legend: When plasma samples were not collected directly at the end of the dosing interval, the concentration (C_p) at the end of the dosing interval (C_{trough}) was extrapolated using historical population half-lives. The TDM pharmacist could also determine if the result was likely subtherapeutic or therapeutic based on expert opinion and by comparing the result to the mean population curve.

Abbreviation: C_{trough}, the concentration at the end of the dosing interval; RAL, raltegravir; t ½, half-life; TDM, therapeutic drug monitoring.

Half-lives references:

1. Bukkems VS, N. Raltegravir once-daily 1200 mg half-life in the second and third trimester in pregnant women living with HIV 14 February 2022 (Personal Communication)
2. Krishna R, East L, Larson P, et al. Effect of metal-cation antacids on the pharmacokinetics of 1200 mg raltegravir. *J Pharm Pharmacol* 2016; 68: 1359-1365. 20160927. DOI: 10.1111/jphp.12632.
3. Moltó J, Valle M, Back D, et al. Plasma and intracellular (peripheral blood mononuclear cells) pharmacokinetics of once-daily raltegravir (800 milligrams) in HIV-infected patients. *Antimicrob Agents Chemother* 2011; 55: 72-75. 20101011. DOI: 10.1128/aac.00789-10.
4. Watts DH, Stek A, Best BM, et al. Raltegravir pharmacokinetics during pregnancy. *J Acquir Immune Defic Syndr* 2014; 67: 375-381. DOI: 10.1097/QAI.0000000000000318.

Supplementary Table 1: Description of subtherapeutic raltegravir therapeutic drug monitoring results and interventions in pregnant women living with HIV

Subject number	Details of subtherapeutic[a] raltegravir concentrations, interventions, and follow-up
#1	RAL 400 mg BID dose - TDM at 22 weeks was considered subtherapeutic by the TDM pharmacist (C_p 0,11 mg/L at 4,5 hours post-dose) and the VL was undetectable. The most probable reason was a suboptimal adherence. The action taken was to encourage adherence.<br><br>The next interpretable TDM was at 30 weeks of gestation with the RAL 400 mg BID dose, and it was therapeutic (C_{trough} = 0.05 mg/L) with an undetectable VL.<br><br>The VL closest to delivery was undetectable.
#2	This woman had two TDM results and both were subtherapeutic.<br><br>RAL HD 1200mg dose – TDM at 25 weeks gestation was subtherapeutic (C_{trough} = 0.01 mg/L) and the VL was undetectable. The most probable reason was suboptimal adherence. The patient was changed to the RAL 400 mg BID dose.<br><br>RAL 400 mg BID dose – TDM at 35 weeks gestation was subtherapeutic (C_p < 0,01 mg/L) and the VL was undetectable. The most probable reason was suboptimal adherence. The action taken was to encourage adherence. The next VL was undetectable.<br><br>The VL closest to delivery was undetectable.
#3	RAL 400 mg BID dose - TDM at 26 weeks gestation was subtherapeutic (C_{trough}= 0.01 mg/L) and the VL was 55 copies /mL. The most probable cause was suboptimal adherence and the action taken was to encourage adherence.<br><br>The next TDM (RAL 400 mg BID dose) was done at 32 weeks gestation and was therapeutic (C_{trough}=0,06 mg/L) and the VL was undetectable.<br><br>The next VL and VL closest to delivery were both undetectable.
#4	RAL 400 mg BID dose - TDM at 35 weeks gestation was subtherapeutic (C_{trough} = 0,01 mg/L) and the VL was undetectable. The most probable cause was unknown, and the action taken was to increase the RAL dose to 600 mg BID. Before this TDM result, the patient had a TDM at 25 weeks gestation taken too early to be interpreted but it was was under the mean general population curve.<br><br>The next VL and VL closest to delivery were both undetectable.
#5	RAL 400 mg BID dose - TDM at 12 weeks gestation was considered subtherapeutic by the TDM pharmacist (C_p 0,13 mg/L at 5 hours post-dose) and the VL was 1112 copies/mL. The most probable cause was unknown, and HIV genotyping was

	requested. No changes to the dose of RAL was done.<br><br>The next TDM (RAL 400 mg BID dose) at 25 weeks gestation was considered therapeutic by the TDM pharmacist (C_p 0,76 mg/L at 2,92 hours post-dose) and the VL undetectable. HIV genotyping was not found.<br><br>The VL closest to delivery undetectable.
#6	This woman had 3 interpretable TDM with the RAL 400 BID dose taken at 16, 25 and 35 weeks of gestation. Only the TDM at 35 weeks of gestation was considered subtherapeutic by the TDM pharmacist (C_p 0.06 mg/L at 4.78 hours post-dose) and the VL was undetectable.<br>The most probable cause was unknown, and the action taken was to encourage adherence.<br><br>The VL closest to delivery was undetectable.
#7	RAL 400 BID dose – TDM at 21 weeks gestation was subtherapeutic (C_p < 0.01mg/L) and the last VL was undetectable. The most probable cause was unknown, and the action taken was to encourage adherence.<br><br>The next VL and the VL closest to delivery were both undetectable.
#8	This woman had six interpretable TDM results of which three were subtherapeutic.<br><br>The first sample considered subtherapeutic (RAL 400 mg BID dose) by the TDM pharmacist (C_p = 0.03 mg/L at 5.42 hours post-dose) was done at 17 week gestation and the VL was undetectable. The most probable cause was unknown, and the action taken was to encourage adherence.<br><br>The second sample considered subtherapeutic (RAL 400 BID dose) by the TDM pharmacist (C_p = 0.05 mg/L at 4.50 hours post-dose) was done at 23 weeks gestation and the VL was undetectable. The most probable cause was suboptimal adherence and the action taken was to increase the RAL dose to 800 mg BID.<br><br>The third sample considered subtherapeutic (RAL 800 BID dose) by the TDM pharmacist (C_p = 0.06 mg/L at 5.92 hours post-dose) was done at 34 weeks gestation and the VL was undetectable. The most probable cause was suboptimal adherence and the action taken was to encourage adherence<br><br>The next TDM (RAL 800 BID dose) was done at 40 weeks gestation and was therapeutic (C_{trough}=0,04 mg/L) and the VL was detectable.<br><br>VL closest to delivery was undetectable.
#9	RAL 400 mg BID dose - TDM at 31 weeks gestation was considered subtherapeutic by the TDM pharmacist (C_p = 0.03 mg/L at 3.08 hours post-dose) and the VL was undetectable. The most probable cause was suboptimal adherence and the action taken was to encourage adherence.

	The next VL and the VL closest to delivery were both undetectable.
#10	RAL 400 mg BID dose - TDM at 36 weeks gestation was considered subtherapeutic by the TDM pharmacist (C_p = 0.25 mg/L at 3.83 hours post-dose) and the VL was undetectable. The most probable cause was a drug interaction between RAL and an antacid.<br>The action taken was to space out the intake of the two drugs by at least 4 hours.<br><br>The next VL and VL closest to delivery were both undetectable.

[a]Threshold used to determine therapeutic status of TDM sample: RAL $C_{trough} \geq 0.02$ mg/L. If the sample was not taken at the end of the dosing interval or a C_{trough} extrapolation was not possible as the sample was taken too early in the dosing interval (i.e., < 8 hours post-dose for twice daily dosing and < 12 hours post-dose for twice daily dosing), the TDM pharmacist could determine if the result was likely subtherapeutic or therapeutic based on expert opinion and by comparing the result to the mean population curve.
Abbreviations: BID, twice daily; C_p, plasma concentration; C_{trough}, concentration at the end of the dosing interval; HD: high dose formulation (600 mg tablet); RAL, raltegravir; TDM, therapeutic drug monitoring; VL, viral load.

Supplementary Table 2: Description of therapeutic drug monitoring results in pregnant women living with HIV with a detectable viral load

Subject number	C_{trough} (mg/L)	Interpretation of RAL TDM	Self-reported adherence in the last 7 days before TDM (%)	Viral load closest to TDM (copies/mL)	ART naïve at conception	Number of days between ART Initiation and VL measure for ART naïve women at conception[a]
#3	0.01	Subtherapeutic	Not adherent [b]	55	No	NA
#11	NR	Therapeutic	NR	113	No	NA
#11	NR	Therapeutic	NR	148	No	NA
#11	NR	Therapeutic	NR	657	No	NA
#11	0.03	Therapeutic	NR	73	No	NA
#5	NR	Subtherapeutic	100	1112	No	NA
#12	NR	Therapeutic	100	4025	No	NA
#6	NR	Therapeutic	NR	74	Yes	71
#8	0.04	Therapeutic	NR	59	No	NA
#13	0.03	Therapeutic	100	99	No	NA
#14	NR	Therapeutic	100	367	No	NA
#15	0.06	Therapeutic	NR	705	Yes	7

[a] Time since initiation of antiretrovirals was not available for women who had started therapy prior to pregnancy.
[b] Patient reported to be not adherent to medication but the number of missing doses was not reported.
Abbreviations: ART: antiretroviral therapy; C_{trough}, concentration at the end of the dosing interval; RAL, raltegravir; NA: not applicable; NR, not reported; TDM, therapeutic drug monitoring; VL, viral load.
